# Influence of the Business Revenue, Recommendation, and Provider Models on Mobile Health App Adoption: Three-Country Experimental Vignette Study

**DOI:** 10.2196/17272

**Published:** 2020-06-04

**Authors:** Francisco Lupiáñez-Villanueva, Frans Folkvord, Mariek Vanden Abeele

**Affiliations:** 1 Departement of Information and Communication Science Universitat Oberta de Catalunya Barcelona Spain; 2 Open Evidence Research Barcelona Spain; 3 Tilburg School of Humanities and Digital Sciences Tilburg University Tilburg Netherlands

**Keywords:** mHealth adoption, experiment, mobile apps, self-monitoring, privacy paradox, business model, data protection, recommendation, health consciousness, health information orientation, eHealth literacy

## Abstract

**Background:**

Despite the worldwide growth in mobile health (mHealth) tools and the possible benefits of mHealth for patients and health care providers, scientific research examining factors explaining the adoption level of mHealth tools remains scarce.

**Objective:**

We performed an experimental vignette study to investigate how four factors related to the business model of an mHealth app affect its adoption and users’ willingness to pay: (1) the revenue model (ie, sharing data with third parties vs accepting advertisements); (2) the data protection model (General Data Protection Regulation [GDPR]-compliant data handling vs nonGDPR-compliant data handling); (3) the recommendation model (ie, doctor vs patient recommendation); and (4) the provider model (ie, pharmaceutical vs medical association provider). In addition, health consciousness, health information orientation, and electronic health literacy were explored as intrapersonal predictors of adoption.

**Methods:**

We conducted an experimental study in three countries, Spain (N=800), Germany (N=800), and the Netherlands (N=416), to assess the influence of multiple business models and intrapersonal characteristics on the willingness to pay and intention to download a health app.

**Results:**

The revenue model did not affect willingness to pay or intentions to download the app in all three countries. In the Netherlands, data protection increased willingness to pay for the health app (*P*<.001). Moreover, in all three countries, data protection increased the likelihood of downloading the app (*P*<.001). In Germany (*P*=.04) and the Netherlands (*P*=.007), a doctor recommendation increased both willingness to pay and intention to download the health app. For all three countries, apps manufactured in association with a medical organization were more likely to be downloaded (*P*<.001). Finally, in all three countries, men, younger individuals, those with higher levels of education, and people with a health information orientation were willing to pay more for adoption of the health app and had a higher intention to download the app.

**Conclusions:**

The finding that people want their data protected by legislation but are not willing to pay more for data protection suggests that in the context of mHealth, app privacy protection cannot be leveraged as a selling point. However, people do value a doctor recommendation and apps manufactured by a medical association, which particularly influence their intention to download an mHealth app.

## Introduction

### Background

Over the last decade, the number of people worldwide who own a mobile phone or another mobile electronic communication device has grown exponentially, fueling the development of mobile health-related services and functions [1,2]. Mobile health (mHealth) [3] can be broadly defined as any medical or public health practice that is supported by mobile devices, ranging from the use of mobile phones to improving points of service data collection, care delivery, and patient communication, to the use of alternative wireless devices for real-time medication monitoring and adherence support (for an overview see [4]). One of the main underlying goals of mHealth is to improve the quality of and access to health care while reducing its costs [5].

Given the potential of mHealth for supporting the health of users, it is important to assess the factors that may motivate or hinder the successful adoption of mHealth technologies and apps. After all, adopting a health technology or app is a first necessary step for ensuring effectiveness [4-6]. However, there is currently insufficient programmatic evidence to inform the implementation and scale-up of mHealth because very little is known about the adoption and effectiveness of mHealth technologies on health [7].

To fill this gap, the aim of this study was to move the field forward by experimentally examining factors that have been suggested to play a role in the adoption of mHealth [8]. We operationalized mHealth adoption in two ways: as having a higher intention to download an mHealth app and being willing to pay a higher price for it. We focus on four factors related to the business model of app development, namely the revenue model, the degree of data protection offered to users, the presence of a doctor recommendation, and whether the app is developed by the pharmaceutical industry or by a medical association. In addition, we explored three intrapersonal characteristics that have been identified as important predictors of electronic health (eHealth) adoption in previous research: health consciousness, health information orientation, and eHealth literacy [9].

Finally, we explored differences among three European countries with varying cultures and health care infrastructures. In Spain, the national health system is an agglomeration of public health services established by the general health law. The vast majority of final providers of care are part of the regional health service structure and are not autonomous legal entities. In Germany, there is a statutory health insurance system that allows people with high incomes to opt out in favor of private coverage. In the Netherlands, there is a statutory health insurance system with universally mandated private insurance (national exchange) that is regulated by the government along with subsidies for insurance. We assume that these differences in national health care infrastructure may impact how users value business models.

### Theoretical Framework

mHealth can serve multiple purposes such as treatment adherence and disease management, smoking cessation, weight loss, diet, and physical activity [10], thereby providing ample opportunities for people to better monitor and manage their personal health with the aid of their smartphone and other wearable devices [8]. In parallel with the rapid development of mHealth technologies, the focus of health care has shifted from health care providers’ paternalistic approach to a more consumer-oriented approach [11]. At the heart of this approach is the belief that allowing patients to actively access their personal health records and manage their own health will encourage them to be more involved in their own health care [12]. This increased involvement can subsequently strengthen the patient-provider relationship and enhance the (cost-) effectiveness of health care management. Because of these individual and societal benefits associated with mHealth, it is important to gain greater insight into business- and person-level factors that may predict its adoption and use.

### mHealth Business Models

A first factor related to the business model that may play a role in mHealth adoption is the revenue model. mHealth operates at the intersection of health, technology, and finance, making it a complex industry for the development of sustainable revenue models [5]. Because consumers do not want to spend a large amount of money on the adoption of health apps [13], a great variety of apps have been developed that make revenue on the basis of advertising; however, personal data are also sold to third parties in some cases. Such apps embrace a revenue model that approaches the “privacy as a product” concept [14]. However, it is likely that people experience having their personal health data sold to third parties as a greater “cost” than merely having to accept advertisements in return for “free” access to and use of an mHealth app, as the security of eHealth data is a major concern in the health care industry [5]. Hence, we established the first hypothesis (H1): people are willing to pay more for a health app (H1a) and have a higher intention to download the app (H1b) when they can access and use the app in exchange for accepting advertisements than when having to accept either data sharing with third parties, or a combination of advertising and data sharing with third parties.

In the arena of health care, previous misuses of patient data have affected public confidence in health care research [15]. This was one of the motivating factors for the European Union to implement the General Data Protection Regulation (GDPR) [16]. The GDPR aims to protect people’s right to protection of their data by establishing rules that are related to the free movement of personal data. The GDPR has received widespread public attention in the public domain, and has led to real and significant changes in the ways in which organizations deal with user data. It is reasonable to assume that the GDPR has sharpened citizens’ awareness of and concern for data protection, including when adopting mHealth apps [17]. Hence, we may expect that adoption of a health app will be positively influenced by assurance of adequate protection of personal health data, leading to hypothesis 2 (H2): people are willing to pay more for a health app (H2a) and have a higher intention to download the app (H2b) when the health app ensures data protection in line with European legislation than when no information is given about data protection.

An additional factor that may play a role in the adoption of mHealth apps is whether the app is recommended by medical professionals, who are considered the gatekeepers of health care delivery [18,19]. As an example, in their analysis of factors affecting the adoption of electronic patient records, Raisinghani and Young [20] noted that doctor recommendations were a key factor in the adoption process. Similarly, Peng et al [21] found that patients with type 2 diabetes identified doctor recommendations as a significant factor motivating their adoption of a diabetes mHealth app [22].

There are at least two reasons to explain why a doctor recommendation for a health app can be a strong enforcer for patients to use digital health technologies. First, doctors are considered to be experts in their field of work, and therefore have more influence than nonexperts, particularly since they also know the patients and their interests quite well [19,23]. Second, doctors’ professionalism forces them to act upon the patients’ interests first; most patients therefore trust a doctor more than other actors [24]. Hence, we devised hypothesis 3 (H3): people are willing to pay more for a health app (H3a) and have a higher intention to download the app (H3b) when the app is recommended by doctors than when the app is recommended by a patient association.

Finally, we examined whether a health app manufactured by a medical association is more likely to be adopted than an app manufactured by the pharmaceutical industry. Pharmaceutical companies need to negotiate the conflict between striving for optimal health care and striving for profit [25]. However, in the eyes of the public, it is not always clear that the pharmaceutical industry has patients’ interests at heart [26].

With the advent of mHealth, new concerns have arisen with regard to the quality of these apps, and whether their development and manufacturing should be regulated [27]. With respect to the implementation of mHealth, there are concerns that when the pharmaceutical industry engages in efforts to disseminate health information via mobile devices, they may strategically use these efforts to promote their products and services [28]. In short, given the for-profit nature of the pharmaceutical industry, we may assume that trust in pharmaceutical providers of mHealth apps is generally lower than trust in providers for whom generating profit is not the main goal, such as medical associations or other nonprofit medical associations. This difference in trust may explain a difference in users’ adoption of mHealth apps, leading to hypothesis 4 (H4): people are willing to pay more for a health app (H4a) and have a higher intention to download the app (H4b) when the app is manufactured by a medical association than when the app is manufactured by a pharmaceutical company.

### Personal Factors Affecting mHealth Adoption: Health Consciousness, Health Information Orientation, and eHealth Literacy

In addition to mHealth business models, we may also consider psychological antecedents that predict adoption [29] to obtain an adequate understanding of personal characteristics that influence the information-use strategies of the online health consumer [30-32]. Studies have shown that the determination to adopt mHealth technologies is greater among people who evaluate their health as more vulnerable to diseases and are more concerned about their health [33], and among people who take more care of their own health [34,35].

According to Dutta-Bergman [34], health consciousness, health information orientation, and eHealth literacy are important factors related to the search for online health information and potentially also to the adoption of a health app. Health consciousness means that an individual takes care of their personal health and that those health concerns are blended into their daily lives [33,36-38]. Health information orientation, defined as the inclination to seek out health information, could be an important predictor to explain who is most willing to adopt a health app [39,40]. Finally, eHealth literacy is considered an important factor predicting health app adoption, since people with higher levels of eHealth literacy have better ability to use health apps [41].

Considering the limited understanding of the general cognitive motivators that trigger people’s usage of health apps, it is important to examine which factors can best explain the adoption of health apps. Therefore, a second aim of this study was to examine whether health consciousness, health information orientation, and eHealth literacy predict the adoption of and willingness to pay for a health app.

## Methods

### Participants and Design

We conducted an online vignette experiment in three countries: Spain, Germany, and the Netherlands. Every participant was exposed to four different vignettes, each describing one specific aspect of the business model of an mHealth app (ie, the first with a specific revenue, the second with data protection, the third with a recommendation, and the fourth with a provider model). Next, the likeliness to adopt the health app and willingness to pay were assessed as outcome measures. For each vignette, a different version was randomly assigned to participants. Vignettes describe a hypothetical situation to which participants respond thereby revealing their perceptions, values, attitudes, and intentions. The advantage of vignette studies is a pragmatic and internal valid method assessing participants’ responses to experimental conditions, thereby simulating actual situations the best way possible. Nonetheless, considering that vignettes are a simulation, actual situations might lead to different outcomes. The revenue model was considered between three subject levels (advertising vs data sharing vs advertising and data sharing), data protection was considered between two subject levels (data protection by European Union legislation vs no information), recommendation was considered between two subject levels (recommended by doctors vs patients association), and provider was considered between two subject levels (medical association vs pharmaceutical company). Table 1 shows the distribution of participants over each vignette condition.

**Table 1:** Number of participants per condition for Spain, Germany, and the Netherlands.

Factors		Spain (N=800)	Germany (N=800)	The Netherlands (N=416)
**Business models**				
	Advertising	268	267	146
	Data sharing	265	266	155
	Advertising and data sharing	267	267	115
**Data protection**				
	European union legislation	400	400	230
	No information provided	400	400	186
**Recommendation**				
	By doctors	400	400	230
	By patients association	400	400	186
**App provider**				
	Medical association	400	400	230
	Pharmaceutical company	400	400	186

The data in Spain (N=800) and Germany (N=800) were collected through an online survey administered by a Spanish professional research company. The sample was chosen through a proportionate stratified sampling method considering gender and age. The data in the Netherlands (N=416) were gathered by snowballing a link of the questionnaire via social media platforms. The participant information is shown in Table 2. Employment status was assessed by the question “Which of these descriptions best describes your situation or applies to what you have been doing for the last month?,” with the answer possibilities ranging from “Employed/Self-employed” to “Another not in the labor force.” We created a bivariate variable with employed vs nonemployed based on this response. Financial status was assessed with the question “During the last 12 months, would you say you had difficulties in paying your bills at the end of the month…?,” with the answer possibilities ranging from “Most of the time” to “Never.” All survey participants were informed of the overall study goals and procedures. Only those who agreed to participate in the online survey were given access to the survey. The approval of the Ethical Committee of the university leading the study (Universitat Oberta de Catalunya, Barcelona, Spain) to conduct the experiment was obtained in 2017. We informed participants beforehand that all of the data collected would remain confidential and that they could cease participation at any time.

**Table 2:** Descriptive information about the participants per country.

Variable		Spain (N=800)		Germany (N=800)		The Netherlands (N=416)	
Gender (women), n (%)		400 (50.0)		400 (50.0)		159 (38.2)	
Age (years), mean (SD)		41.60 (13.32)		45.92 (15.09)		28.92 (14.86)	
**Education level, n (%)**							
	Primary education	26 (3.3)		224 (28.0)		8 (1.9)	
	High school diploma	200 (25.0)		285 (35.6)		57 (13.7)	
	Some years of university	136 (17.0)		59 (7.4)		105 (25.2)	
	University	335 (41.9)		170 (21.3)		175 (42.1)	
	Postgraduate degree	103 (12.9)		62 (7.8)		71 (17.1)	
**Employment status, n (%)**							
	Employed/self-employed	586 (73.3)		468 (58.5)		161 (38.7)	
	Unemployed	64 (8.0)		36 (4.5)		9 (2.2)	
	Student	55 (6.9)		55 (6.9)		238 (57.2)	
	Retired	60 (7.5)		178 (22.3)		4 (1.0)	
	Not working due to illness or disability	12 (1.5)		27 (3.4)		2 (0.5)	
	Another not in the labor force	23 (2.9)		36 (4.5)		2 (0.5)	
**Financial status^a^, n (%)**							
	Most of the time	123 (15.4)		62 (7.8)		21 (5.0)	
	From time to time	293 (36.6)		199 (24.9)		78 (18.8)	
	Almost never	375 (46.9)		525 (65.6)		291 (70.0)	
	No answer	9 (1.1)		14 (1.8)		26 (6.3)	
**Health app use, n (%)**							
	No use	420 (52.5)		565 (70.6)		285 (68.5)	
	One time	105 (13.1)		71 (8.9)		37 (8.9)	
	Two times	82 (10.3)		73 (9.1)		28 (6.7)	
	Three times	71 (8.9)		36 (4.5)		25 (6.0)	
	Four times	33 (4.1)		10 (1.3)		11 (2.6)	
	Five times	22 (2.8)		13 (1.6)		4 (1.0)	
	More than six times	67 (8.4)		32 (4.0)		26 (6.3)	
Health consciousness, mean (SD)		4.06 (0.78)		3.82 (0.85)		3.43 (0.87)	
Health information orientation, mean (SD)		3.63 (0.83)		3.30 (0.96)		2.79 (1.01)	
eHealth^b^ literate, mean (SD)		3.51 (0.93)		3.50 (0.94)		3.08 (1.03)	
**Doctor visits in the last year**							
	Never	78 (9.8)		95 (11.9)		102 (24.4)	
	Once	156 (19.5)		114 (14.2)		87 (20.9)	
	Twice	170 (21.3)		151 (18.9)		80 (19.2)	
	Three times	128 (16.0)		104 (13.0)		54 (13.0)	
	Four times	86 (10.8)		97 (12.1)		31 (7.5)	
	Five times or more	182 (22.8)		236 (29.5)		57 (13.7)	
**General health, n (%)**							
	Very bad	1 (0.1)		11 (1.4)		143 (34.4)	
	Bad	21 (2.6)		78 (9.8)		223 (53.6)	
	Neither good nor bad	129 (16.1)		204 (25.5)		41 (9.9)	
	Good	461 (57.6)		391 (48.9)		6 (1.4)	
	Very good	188 (23.5)		116 (14.5)		3 (0.7)	

^a^Based on the response to the question “During the last 12 months, would you say you had difficulties in paying your bills at the end of the month…?”.

^b^eHealth: electronic health.

### Procedure

When individuals agreed to participate in the study, they answered a series of demographic questions (gender, age, education, employment status). The participants were then presented with four vignettes for the revenue, data protection, recommendation, and provider model. For example, the vignette for the revenue model stated:

Imagine that an app is presented to you to support you in improving the healthiness of your lifestyle by recording your personal data (for example, nutritional intake, physical behavior, heart rate, glucose level, calories burnt, etc), providing prescriptions and consultations, and checking your health history. Based on your collected data, the app will provide tailored advice to improve your health. Revenues of this health app come from ads shown to you when using the app. We want you, on an as-honestly-as-possible basis, to evaluate how much you want to pay for the health app, if you were to buy it in an app store.

The participants were then asked about their willingness to pay and their intention to download the app. Finally, the participants answered general questions relating to health app usage, health consciousness, health information orientation, health literacy, and health issues.

### Measures

#### Dependent Variables

Willingness to pay was measured through responses to the open question “What is the highest price you are willing to pay?” (in Euros). Willingness to download the app was measured with the question “Please indicate on a scale of 1 to 10 how likely it is that you would download the app?” (1=definitely not download the app, 10=definitely download the app).

#### Intrapersonal Factors

Health app usage was measured by asking how often the participant used a health app, varying from 0 (never) to 6 (more than 5 times), and how much time the participant spent using a health app in the last week, varying from 0 (0 hours) to 6 (more than 1 hour).

Health consciousness was measured using 5 statements that were each rated on a 5-point scale (1 strongly disagree to 5 strongly agree) [39]. Reliability of the scale was high (Cronbach α=.88). Health information orientation was measured using 8 statements each rated on a 5-point scale (1 strongly disagree to 5 strongly agree) [39]. Reliability of the scale was high (Cronbach α=.93) eHealth literacy was measured using 8 statements each rated on a 5-point scale (1 strongly disagree to 5 strongly agree) [42]. Reliability of the scale was high (Cronbach α=.95).

### Statistical Analyses

Multiple linear regression models were conducted for every country and the two dependent variables (willingness to pay and intention to download) separately. Within each regression, in the first step we assessed the effect of the business model; in the second step we included age, gender, and education; and in the third step we included health consciousness, health information orientation, and eHealth literacy. In addition, effect sizes were calculated for each regression model. Following Cohen [43], an effect size of *R^2^* around 0.1 is interpreted as low, *R^2^* varying around 0.3 is considered medium, and *R^2^*>0.5 is interpreted as a large effect.

## Results

### Revenue Models

Linear regression analyses were first conducted to explore the role of the revenue model for Spain (see Multimedia Appendix 1). We first examined if Spanish people were more willing to pay more for a health app when they could access and use the app in exchange for accepting advertisements than when having to accept data sharing with third parties, either individually or with a combination of advertising (H1a). No effects were found for data sharing (*P=*.20) or data sharing and advertising (*P=*.50) as revenue models (advertising was used as the reference category) on willingness to pay. Furthermore, men (*P=*.02) and people with a health information orientation (*P=*.002) were more willing to pay more for the health app. The explained variance for the model including all predictors was 3.2%. Next, we tested the same model but with intention to download the app as the outcome measure (H1b). Again, no effects were found for data sharing (*P=*.95) or data sharing and advertising (*P=*.19) as business models on the intention to download in all three models. Men (*P=.*005), younger people (*P<.*001), those employed (*P=.*002), and people with a health information orientation (*P<.*001) reported greater intentions to download the health app. The explained variance for the model including all predictors was 22.4%. These findings do not support H1a and H1b.

Similar results were obtained in the analyses for the German sample (see Multimedia Appendix 2). No effects were found for data sharing (*P=.*23) or data sharing and advertising (*P=.*07) as revenue models (advertising as the reference category) on willingness to pay (H1a) in all three models. Furthermore, younger people (*P*=.02), people who obtained a postgraduate degree (*P=*.01) compared to students, those who were employed (*P*=.02), and people with a health information orientation (*P*<.001) were more willing to pay more for adopting the health app. The explained variance for the model including all predictors was 7.94%. Next, we tested the same model but with intention to download the app as the outcome measure (H1b). Again, no effects were found for data sharing (*P*=.26) or data sharing and advertising (*P*=.08) as revenue models on the intention to download in all three models. Men (*P*=.02), younger people (*P*<.001), people who finished high school (*P*=.01) or university (*P*=.003), those who were employed (*P<.*001), and people with a health information orientation (*P*<.001) reported greater intentions to download the health app. The explained variance for the model including all predictors was 29.7%. These findings do not support H1a and H1b.

Similar results were also obtained for the Netherlands (see Multimedia Appendix 3). No effects were found for data sharing (*P*=.38) or data sharing and advertising (*P*=.17) as revenue models (advertising as the reference category) on willingness to pay in all models. Furthermore, men (*P*=.02), those who were employed (*P=.*01), and people with a health information orientation (*P*=.05) were willing to pay more for adopting the health app. The explained variance for the model including all predictors was 5.3%. For intentions to download the app, no effects were found for data sharing (*P*=.38) or data sharing and advertising (*P*=.43) as revenue models in all three models. People with a health information orientation had greater intentions to download the health app (*P*=.05). The explained variance for the model including all predictors was 3.5%. These findings do not support H1a and H1b.

### Data Protection Models

Next, we explored the role of the data protection model. Linear regression analyses were first performed for Spain (see Multimedia Appendix 4) to examine if people were willing to pay more for a health app (H2a) and had a higher intention to download the app (H2b) when the health app ensured data protection in line with European legislation than when no information was given about data protection. No effects were found for the data protection model (no information about data protection was the reference category) on willingness to pay in all three models. Furthermore, men (*P*=.03) and people with a health information orientation (*P*=.006) were willing to pay more for adopting the health app. The explained variance for the model including all predictors was 3.0%. In contrast, participants in the condition whereby data were protected by European Union legislation had greater intentions to download the app (*P*<.001) in all three models. Furthermore, men (*P*=.01), younger people (*P*<.001), and people with a health information orientation (*P*<.001) had greater intention to download the health app. The explained variance for the model including all predictors was 23.3%. These results do not support H2a but do support H2b.

Next, we conducted the linear regression analyses for Germany (see Multimedia Appendix 5). Again, no effects were found for the data collection model (*P*=.08) on willingness to pay in all three models. People with a health information orientation (*P*<.001) and with less eHealth literacy (*P*=.03) were willing to pay more for the health app. The explained variance for the model including all predictors was 4.8%. In contrast, participants in the condition whereby data were protected by European Union legislation had greater intentions to download the app (*P*<.001) in all three models. Furthermore, men (*P*=.006); younger people (*P*<.001); people who finished high school (*P*=.001), university (*P*<.001), or had a postgraduate degree (*P*=.03); and people with a health information orientation (*P*<.001) reported greater intentions to download the health app. The explained variance for the model including all predictors was 33.6%. These results do not support H2a but do support H2b.

Finally, linear regression analyses were conducted for the Netherlands (see Multimedia Appendix 6). Participants in the condition whereby data protection by European Union legislation was explicitly stated were more willing to pay more for the health app than participants who received no information about data protection (*P*<.001). No significant effects were found for the other factors. The explained variance for the model including all predictors was 5.7%. In addition, participants in the condition where data were protected by European Union legislation reported greater intentions to download (*P*<.001) in all three models. Furthermore, people with a health information orientation had a greater intention to download the health app (*P*=.003). The explained variance for the model including all predictors was 11.2%. These results support both H2a and H2b.

### Recommendation Models

Next, we explored the role of the recommendation model. The first linear regression analyses were conducted for Spain (see Multimedia Appendix 7) to examine if people were more willing to pay more for a health app (H3a) and had a higher intention to download the app (H3b) when the app was recommended by doctors than when the app was recommended by a patient association (reference category). No effects were found for the recommendation model on willingness to pay in all three models. Furthermore, men (*P*=.02) and people with a health information orientation (*P*=.01) were willing to pay more for adopting the health app. The explained variance for the model including all predictors was 3.4%. In contrast, participants reported greater intentions to download the health app when doctors recommended the health app (*P*=.04) compared to when the patients association recommended the health app. Furthermore, men (*P*=.02), younger people (*P*<.001), those who were employed (*P=.*01), and people with a health information orientation (*P*<.001) reported greater intentions to download the health app. The explained variance for the model including all predictors was 21.2 %. These results do not support H3a but do support H3b.

Next, we conducted linear regression analyses for Germany (see Multimedia Appendix 8). Participants were more willing to pay for the app when it was recommended by doctors than when it was recommended by the patients association (*P*=.02), except in the model including all predictors. Furthermore, people who finished university (*P*=.007) compared to students, and people with a health information orientation (*P*<.001) were willing to pay more for adopting the health app. In contrast, people with more eHealth literacy were less willing to pay more for the health app (*P*=.007). The explained variance for the model including all predictors was 7.6%. Participants had greater intentions to download the health app when it was recommended by doctors compared to when it was recommended by the patients association (*P*=.01), but only when not controlling for sociodemographic and dispositional factors. Furthermore, men (*P*=.02); younger people (*P*<.001); people who finished high school (*P*=.001) and university (*P*<.001), or those with a postgraduate degree (*P*=.009) compared to students; those who were employed (*P*<.001); and people with a health information orientation had greater intention to download the health app (*P*<.001). The explained variance for the model including all predictors was 31.2%. These results support both H3a and H3b.

Finally, linear regression analyses were conducted for the Netherlands (see Multimedia Appendix 9). Participants were willing to pay more for the health app when it was recommended by doctors compared to when it was recommended by the patients association (*P*=.01) in all three models. No significant effect was found for the other factors. The explained variance for the model including all predictors was 2.7%. In addition, participants reported greater intentions to download the health app when it was recommended by doctors compared to when it was recommended by the patients association (*P*=.01). Furthermore, people with a health information orientation had greater intentions to download the health app (*P*<.001). The explained variance for the model including all predictors was 8.6%. These results support both H3a and H3b.

### Provider Models

Finally, we conducted linear regression analyses to explore the role of the provider model. The first set of analyses were conducted for Spain (see Multimedia Appendix 10). We examined if people were more willing to pay more for a health app (H4a) and had a higher intention to download the app (H4b) when the app was manufactured by a medical association than when it was manufactured by a pharmaceutical company (reference category). No effects were found for the provider model on willingness to pay in all three models. Furthermore, men (*P*=.03) and people with a health information orientation (*P*=.004) were willing to pay more for adopting the health app. The explained variance for the model including all predictors was 3.5%. In contrast, participants had less intention to download the health app if it was provided by a medical association compared to when it was provided by a pharmaceutical company (*P*<.001) in all three models. Furthermore, men (*P*=.006), younger people (*P*<.001), those who were employed (*P*=.009), and people with a health information orientation (*P*<.001) had greater intentions to download the health app. The explained variance for the model including all predictors was 23.1%. These results do not support H4a but do support H4b.

The second set of linear regression analyses were conducted for Germany (see Multimedia Appendix 11). No significant effects were found for the provider model on willingness to pay in all three models. Furthermore, younger people (*P*=.005), people with a health information orientation (*P*<.001), and people with less eHealth literacy (*P*=.002) were willing to pay more for adopting the health app. The explained variance for the model including all predictors was 6.3%. In contrast, participants reported less intentions to download the health app when it was provided by a medical association compared to when it was provided by a pharmaceutical company (*P*<.001) in all three models. Furthermore, men (*P*=.002); younger people (*P*<.001); people who finished high school (*P*=.005), university (*P*<.001), or had a postgraduate degree (*P*=.03), with students as reference; people with a health information orientation (*P*<.001); and people with less eHealth literacy (*P*=.02) had greater intentions to download the health app. The explained variance for the model including all predictors was 29.9%. These results do not support H4a but do support H4b.

Finally, linear regression analyses were conducted for the Netherlands (see Multimedia Appendix 12). Participants were willing to pay more for the health app when it was provided by a medical association compared to when it was provided by a pharmaceutical company (*P*=.005) in all three models. No significant effects were found for the other factors. The explained variance for the model including all predictors was 5.0%. In addition, participants reported greater intentions to download the app when it was provided by a medical association than when it was provided by a pharmaceutical company (*P*<.001) in all three models. Furthermore, people with a health information orientation had greater intention to download the health app (*P*=.008). The explained variance for the model including all predictors was 7.0%. These results support both H4a and H4b.

## Discussion

### Principal Findings

Given the expected benefits associated with mHealth adoption, both for individual users and health care systems, it is important to gain greater understanding of factors that contribute to or deter from adoption. Therefore, we conducted an online experiment to assess the effect of four variations in the business model of an mHealth app and three intrapersonal characteristics in three different countries (Spain, Germany, and the Netherlands) on individuals’ willingness to pay for and their likelihood of adopting an mHealth app.

The results showed that in all countries there was no effect of the different revenue models on both willingness to pay and intention to download the health app, thereby not supporting H1. People are not less willing to pay and do not have a reduced intention to download a health app when the revenue model is based on data sharing or advertising and data sharing, compared to that based on advertising only. This finding is surprising, as people in general report being concerned about sharing their personal information [42]. Hence, this concern could be expected to drive one’s intended and actual disclosure, and their subsequent decision making. Our study does not support this speculation, and instead suggests that people are less than selective and often cavalier in the protection of their own data profiles. To date, few studies have examined this discrepancy between individuals’ intentions to protect their own privacy and how they actually behave in the marketplace, which is termed the “privacy paradox” (see [42]) in the context of mHealth. Our findings indicate that further research on this matter is warranted, given that the privacy paradox is an increasing concern when it comes to personal health data [11,44].

Interestingly, in Spain and Germany, we found no effects of the data protection model on willingness to pay, whereas in the Netherlands, participants in the data protection condition were willing to pay more for the health app compared to when receiving no information regarding on how their health information will be used. In all three countries, participants in the data protection condition were more likely to download the health app, thereby partly supporting H2. Thus, in line with the findings for the revenue model, and supporting the notion of the privacy paradox, people were not willing to pay for the app. Given an industry in which mobile apps are continuously expanding and new health care apps and devices are rapidly being created, it is essential to be very cautious of the collection and treatment of users’ personal health information, particularly by the consumers themselves [44].

In Spain, we found no effect of the recommendation model, whereas in Germany and the Netherlands, participants were willing to pay more for a health app recommended by a doctor compared to that recommended by a patient association. In addition, in all three countries, intentions to download the health app were greater when the app was recommended by a doctor.

In Spain and Germany, we found no effects of the provider model, whereas in the Netherlands, participants in the medical association provider condition were willing to pay more for the health app than participants in the pharmaceutical provider condition. In all three countries, participants in the medical association provider condition had greater intentions to download the app than participants in the pharmaceutical provider condition.

Overall, the findings of our study indicate that endorsement from the medical establishment, either via a doctor recommendation or a medical association provider model, is helpful to increase adoption of an mHealth app. However, the revenue and data protection models seem to have a less consistent and a weaker effect, especially on the willingness to pay for an app. These findings suggest that future app developers can benefit most from a close collaboration with medical experts and organizations to increase adoption rates.

In general, the above findings show that certain aspects of the business model can influence the willingness to pay for or the intention to adopt an mHealth app, but that this influence appears conditional; that is, it varies according to the country of residence and seems to interact with dispositional characteristics such as a person’s health information orientation. In summary, this suggests that mHealth adoption is a complex process that involves many different factors situated at least at the personal, economic, and cultural level. This implies that in order to increase adoption rates and decrease attrition, developers, organizations, and practitioners need to be weary of one-size-fits-all approaches, as these are likely less successful than an approach that tailors the business model to the population of interest. Given that we currently lack understanding of the precise mechanisms that explain why, under certain conditions, mHealth adoption can be more or less successful, future research is needed to explore these mechanisms in greater depth.

Finally, in all three countries, men, younger individuals, people with higher levels of education, and those with a health information orientation were willing to pay more for adoption of the health app and had a higher intention to download the app. In line with previous studies, health information orientation was found to be an important predictor that explains both the willingness to pay and the intention to download the health app [36,39,40]. A high level of health information orientation positively affected the amount a participant was willing to pay for the health app and the intention to download it.

Overall, the finding that young, highly educated males, and people with a stronger health information orientation were more willing to pay for and download the mHealth app in this study suggests that traditional factors that demarcate access to and use of health services such as gender and age are also at play in mHealth. Owing to the ease of use and widespread diffusion of mobile phones, mHealth initiatives are often applauded for their emancipatory potential (eg, [45]); thus, our study supports earlier observations that future policy efforts aimed at closing “the digital health divide” need to also focus on disparities in mHealth adoption and use [46]. To inform these policy efforts, further research is needed to explore the specific barriers hindering participation in mHealth.

### Strengths and Limitations

One of the strengths of the current study is that we collected data among a large group of participants in three different countries. Another strength is that we used a multifactorial experimental design, examining several factors in relation to the business model that are considered to be important in predicting and explaining the adoption of a health app. Third, we assessed the role of three intrapersonal predictors of the adoption of a health app.

This study also had some limitations. First, because the study was conducted online, internal validity of the exact experiment cannot be guaranteed since it is difficult to assess how truthfully the participants answered. Nonetheless, because the experiment was not focused on sensitive questions but rather on factors related to adoption of an online health app, using an online questionnaire to assess different factors could be considered a valid and reliable measurement. Second, both in Spain and Germany, data were collected by a professional company in which the participants were paid for their participation, whereas in the Netherlands a convenience sampling approach was used without paying the participants. Overall, the results are quite similar between the countries, although we also noticed some minor differences in some results that could be due to the different sampling methods.

### Conclusion

Over the last decade, the number of people in the world who perform health-related functions on their smartphones has increased rapidly [1,2]. However, research into the adoption and effectiveness of mHealth remains scarce. This is unfortunate, given that adopting a health app is a necessary first step for such an app to be effective [6,44,47]. It is essential that patient safety (data protection), reducing costs, and creating sound business models are investigated to a larger extent to gain a better understanding of the major driving forces for the adoption of mHealth in the future. Next, it is important to create standards for mobile apps, whereby doctors and patients associations can have a leading role in informing the potential consumers as a heuristic approach. Governments, large funders, and industry associations should create and adhere to such standards so that mHealth apps can be adopted and used with confidence of the quality, privacy of the data, and with prices that are proportional to the service provided.

## Appendix

Multimedia Appendix 1 Linear regression analyses with willingness to pay and intention to download for the business models in Spain.

Multimedia Appendix 2 Linear regression analyses with willingness to pay and intention to download for the business models in Germany.

Multimedia Appendix 3 Linear regression analyses with willingness to pay and intention to download for the business models in the Netherlands.

Multimedia Appendix 4 Linear regression analyses with willingness to pay and intention to download for the data collection models in Spain.

Multimedia Appendix 5 Linear regression analyses with willingness to pay and intention to download for the data collection models in Germany.

Multimedia Appendix 6 Linear regression analyses with willingness to pay and intention to download for the data collection models in the Netherlands.

Multimedia Appendix 7 Linear regression analyses with willingness to pay and intention to download for the recommendation models in Spain.

Multimedia Appendix 8 Linear regression analyses with willingness to pay and intention to download for the recommendation models in Germany.

Multimedia Appendix 9 Linear regression analyses with willingness to pay and intention to download for the recommendation models in the Netherlands.

Multimedia Appendix 10 Linear regression analyses with willingness to pay and intention to download for the provider models in Spain.

Multimedia Appendix 11 Linear regression analyses with willingness to pay and intention to download for the provider models in Germany.

Multimedia Appendix 12 Linear regression analyses with willingness to pay and intention to download for the provider models in the Netherlands.

## Abbreviations

eHealth: electronic health
GDPR: General Data Protection Regulation
H1: hypothesis 1
H2: hypothesis 2
H3: hypothesis 3
mHealth: mobile health
