# Supplementary material for: Influence of the Business Revenue, Recommendation, and Provider Models on Mobile Health App Adoption: Three-Country Experimental Vignette Study
Source: JMIR Mhealth Uhealth. 2020 Jun 4;8(6):e17272. doi: 10.2196/17272 (PMC7303831; doi:10.2196/17272)
Supplement: Multimedia Appendix 1 [file mhealth_v8i6e17272_app1.docx]

Multimedia Appendix 1. Linear regression analyses with willingness to pay and intention to download for the business models in Spain^1^

|  | Spain | | | | | |
| --- | --- | --- | --- | --- | --- | --- |
|  | WTP | | | Intention to Download | | |
|  | Model 1^3^ | Model 2 | Model 3 | Model 1 | Model 2^3^ | Model 3^3^ |
| Constant | **3.221 (.000)** | 2.360 (.280) | **2.633** (.215) | **6.239 (.000)** | **8.024 (.000)** | **3.694 (.000)** |
| Business model (advertising is ref)  Data sharing  Data sharing and Advertising | 0.755 (.200)  0.395 (.502) | 0.856 (.149)  0.378 (.522) | 0.177 (.763)  0.790 (.179) | 0.014 (.950)  0.289 (.193) | 0.011 (.959)  0.221 (.307) | -0.054 (.787)  -0.111 (.578) |
| Gender (male is ref) |  | **-1.108 (.023)** | **-1.159 (.017)** |  | **-0.426 (.017)** | **-0.522 (.002)** |
| Age |  | -0.013 (.497) | -0.003 (.871) |  | **-0.032 (.000)** | **-0.026 (.000)** |
| Education (student is ref)  High school  Some university  University  Postgraduate |  | -0.204 (.886)  0.060 (.474)  0.836 (.410)  1.263 (.406) | -0.228 (.873)  0.289 (.844)  0.998 (.478)  1.273 (.401) |  | -0.428 (.414)  -0.853 (.115)  -0.333 (.519)  -0.158 (.777) | -0.614 (.203)  -0.709 (.155)  -0.328 (.491)  -0.301 (.558) |
| Employed (yes is ref)  Financial status (mostly is ref) |  | 0.275 (.634) | 0.132 (.820) |  | **0.668 (.002)** | **0.451 (.022)** |
| From time to time  Almost never |  | 0.358 (.626)  0.174 (.804) | 0.378 (.607)  0.234 (.741) |  | 0.460 (.088)  -0.057 (.826) | 0.479 (.054)  -0.063 (.792) |
| Health consciousness |  |  | -.583 (.149) |  |  | 0.035 (.796) |
| Health information orientation |  |  | **1.363 (.002)** |  |  | **1.099 (.000)** |
| eHealth literacy |  |  | -0.199 (.571) |  |  | 0.116 (.330) |
| *Effect size (R^2^*) | *0.002* | *0.018* | *0.032* | *0.003* | *0.077* | *.224* |

^1^ N= 800

^2^ *P* < .05

^3^ *P* < .01
