# Supplementary material for: Influence of the Business Revenue, Recommendation, and Provider Models on Mobile Health App Adoption: Three-Country Experimental Vignette Study
Source: JMIR Mhealth Uhealth. 2020 Jun 4;8(6):e17272. doi: 10.2196/17272 (PMC7303831; doi:10.2196/17272)
Supplement: Multimedia Appendix 2 [file mhealth_v8i6e17272_app2.docx]

Multimedia Appendix 2. Linear regression analyses with willingness to pay and intention to download for the business models in Germany^1^

|  | Germany | | | | | |
| --- | --- | --- | --- | --- | --- | --- |
|  | WTP | | | Intention to Download | | |
|  | Model 1 | Model 2^3^ | Model 3^3^ | Model 1 | Model 2^3^ | Model 3^3^ |
| Constant | **3.273 (.000)** | **4.622 (.000)** | 2.231 (.087) | **5.247 (.000)** | **5.890 (.000)** | **2.417 (.000)** |
| Business model (advertising is ref)  Data sharing  Data sharing and Advertising | -0.553 (.225)  -0.839 (.066) | -0.440 (.451)  -0.743 (.099) | -0.528 (.235)  -0.717 (.106) | -0.304 (.257)  -0.468 (.080) | -0.208 (.415)  -0.354 (.163) | -0.296 (.196)  -0.309 (.174) |
| Gender (male is ref) |  | -0.175 (.635) | -0.328 (.368) |  | **-0.468 (.020)** | **-0.692 (.000)** |
| Age |  | **-0.030 (.021)** | **-0.031 (.015)** |  | **-0.034 (.000)** | **-0.036 (.000)** |
| Education (student is ref)  High school  Some university  University  Postgraduate  Employed (yes is ref)  Financial Status (mostly is ref)  From time to time  Almost never |  | 0.161 (.735)  0.844 (.275)  0.961 (.077)  **1.955 (.010)**  **0.957 (.015)**  -0.743 (.292)  -0.939 (.148) | 0.004 (.994)  0.541 (.478)  0.705 (.192)  **1.759 (.020)**  0.593 (.133)  -0.923 (.184)  -1.127 (.079) |  | **0.673 (.012)**  0.394 (.366)  **0.921 (.003)**  0.498 (.246)  **1.176 (.000)**  0.620 (.120)  0.364 (.321) | 0.455 (.060)  -0.021 (.957)  0.524 (.059)  0.186 (.630)  **0.657 (.001)**  0.361 (.311)  0.086 (.793) |
| Health consciousness |  |  | 0.106 (.704) |  |  | 0.067 (.638) |
| Health information orientation |  |  | **1.163 (.000)** |  |  | **1.437 (.000)** |
| eHealth literacy |  |  | -0.292 (.261) |  |  | -0.108 (.418) |
| *Effect size (R^2^*) | *0.004* | *0.044* | *0.079* | *0.004* | *0.082* | *0.297* |

^1^ N= 800

^2^ *P* < .05

^3^ *P* < .01
