# Supplementary material for: Influence of the Business Revenue, Recommendation, and Provider Models on Mobile Health App Adoption: Three-Country Experimental Vignette Study
Source: JMIR Mhealth Uhealth. 2020 Jun 4;8(6):e17272. doi: 10.2196/17272 (PMC7303831; doi:10.2196/17272)
Supplement: Multimedia Appendix 3 [file mhealth_v8i6e17272_app3.docx]

Multimedia Appendix 3

Linear regression analyses with willingness to pay and intention to download for the business models in the Netherlands

|  | The Netherlands | | | | | |
| --- | --- | --- | --- | --- | --- | --- |
|  | WTP | | | Intention to Download | | |
|  | Model 1 | Model 2^2^ | Model 3 | Model 1 | Model 2 | Model 3 |
| Constant | **4.188 (.000)** | 4.024 (.098) | 4.097 (.215) | **4.555 (.000)** | **4.663 (.000)** | **4.285 (.001)** |
| Business model (advertising is ref)  Data sharing  Data sharing and Advertising | -0.727 (.381)  -1.227 (.169) | -0.232 (.782)  -1.188 (.183) | -0.498 (.557)  -1.156 (.194) | -0.264 (.380)  -0.259 (.426) | -0.218 (.487)  -0.230 (.479) | -0.291 (.500)  -0.222 (.349) |
| Gender (male is ref) |  | **-1.740 (.019)** | **-1.796 (.015)** |  | -0.376 (.165) | -0.395 (.145) |
| Age |  | -0.023 (.354) | -0.024 (.338) |  | -0.016 (.098) | -0.015 (.110) |
| Education (student is ref)  High school  Some university  University  Postgraduate  Employed (yes is ref)  Financial Status (mostly is ref)  From time to time  Almost never |  | 2.363 (.361)  3.863 (.133)  1.802 (.475)  1.206 (.641)  **1.999 (.014)**  -0.358 (.786)  -0.556 (.746) | 1.841 (.479)  3.300 (.201)  1.367 (.588)  0.939 (.716)  **1.889 (.020)**  -0.275 (.835)  -0.365 (.746) |  | 1.471 (.140)  1.105 (.265)  0.854 (.379)  1.121 (.259)  0.024 (.936)  -0.080 (.870)  -0.131 (.756) | 1.235 (.217)  0.869 (.381)  0.702 (.470)  0.999 (.314)  -0.041 (.890)  -0.003 (.996)  -0.022 (.958) |
| Health consciousness |  |  | -0.060 (.901) |  |  | -0.123 (.488) |
| Health information orientation |  |  | **0.917 (.047)** |  |  | **0.336 (.047)** |
| eHealth literacy |  |  | -0.321 (.409) |  |  | 0.004 (.975) |
| *Effect size (R^2^*) | *0.005* | *0.053* | *0.066* | *0.002* | *0.022* | *0.035* |

^1^ N= 380

^2^ *P* < .05

^3^ *P* < .01
