# Supplementary material for: Influence of the Business Revenue, Recommendation, and Provider Models on Mobile Health App Adoption: Three-Country Experimental Vignette Study
Source: JMIR Mhealth Uhealth. 2020 Jun 4;8(6):e17272. doi: 10.2196/17272 (PMC7303831; doi:10.2196/17272)
Supplement: Multimedia Appendix 4 [file mhealth_v8i6e17272_app4.docx]

Multimedia Appendix 4

Linear regression analyses with willingness to pay and intention to download for the data collection models in Spain

|  | Spain | | | | | |
| --- | --- | --- | --- | --- | --- | --- |
|  | WTP | | | Intention to Download | | |
|  | Model 1 | Model 2 | Model 3^1^ | Model 1^3^ | Model 2^3^ | Model 3 |
| Constant | **3.679 (.000)** | **4.822 (.010)** | 2.672 (.223) | **7.560 (.000)** | **9.176 (.000)** | **4.680 (.000)** |
| Data collection models (no protection is ref) | 0.265 (.559) | 0.141 (.759) | 0.087 (.849) | **0.972 (.000)** | **0.897 (.000)** | **0.813 (.000)** |
| Gender (male is ref) |  | **-0.989 (.032)** | **-1.044 (.023)** |  | **-0.463 (.013)** | **-0.564 (.001)** |
| Age |  | -0.014 (.426) | -0.004 (.812) |  | **-0.037 (.000)** | **-0.031 (.000)** |
| Education (student is ref)  High school  Some university  University  Postgraduate  Employed (yes is ref)  Financial Status (mostly is ref)  From time to time  Almost never |  | -0.306 (.820)  -0.155 (.911)  0.409 (.758)  1.162 (.418)  0.499 (.362)  0.401 (.561)  0.032 (.962) | -0.323 (.810)  0.039 (.978)  0.544 (.682)  1.132 (.429)  0.357 (.515)  0.445 (.520)  0.122 (.855) |  | 0.056 (.918)  -0.037 (.599)  0.144 (.788)  0.333 (.564)  **0.616 (.005)**  0.323 (.245)  -0.216 (.418) | -0.108 (.831)  -0.274 (.599)  0.167 (.738)  0.188 (.726)  **0.409 (.047)**  0.337 (.195)  -0.216 (.389) |
| Health consciousness |  |  | -.581 (.127) |  |  | 0.025 (.859) |
| Health information orientation |  |  | **1.151 (.006)** |  |  | **1.044 (.000)** |
| eHealth literacy |  |  | -0.034 (.918) |  |  | 0.150 (.227) |
| *Effect size (R^2^*) | *0.000* | *0.017* | *0.030* | *0.032* | *0.107* | *0.233* |

^1^ N= 800

^2^ *P* < .05

^3^ *P* < .01
