# Supplementary material for: Influence of the Business Revenue, Recommendation, and Provider Models on Mobile Health App Adoption: Three-Country Experimental Vignette Study
Source: JMIR Mhealth Uhealth. 2020 Jun 4;8(6):e17272. doi: 10.2196/17272 (PMC7303831; doi:10.2196/17272)
Supplement: Multimedia Appendix 5 [file mhealth_v8i6e17272_app5.docx]

Multimedia Appendix 5

Linear regression analyses with willingness to pay and intention to download for the data collection models in Germany

|  | Germany | | | | | |
| --- | --- | --- | --- | --- | --- | --- |
|  | WTP | | | Intention to Download | | |
|  | Model 1 | Model 2^2^ | Model 3^3^ | Model 1^3^ | Model 2^3^ | Model 3^3^ |
| Constant | **3.686 (.000)** | **2.950 (.018)** | 1.365 (.340) | **6.568 (.000)** | **7.208 (.000)** | **3.399 (.000)** |
| Data collection models (no protection is ref) | 0.639 (.081) | 0.628 (.086) | 0.556 (.126) | **1.105 (.000)** | **1.108 (.000)** | **0.967 (.000)** |
| Gender (male is ref) |  | 0.086 (.815) | -0.032 (.931) |  | **-0.576 (.006)** | **-0.795 (.000)** |
| Age |  | -0.018 (.157) | -0.019 (.140) |  | **-0.033 (.000)** | **-0.036 (.000)** |
| Education (student is ref)  High school  Some university  University  Postgraduate  Employed (yes is ref)  Financial Status (mostly is ref)  From time to time  Almost never |  | 0.682 (.150)  1.045 (.173)  0.990 (.067)  0.713 (.346)  0.411 (.292)  0.655 (.350)  0.678 (.294) | 0.578 (.218)  0.827 (.277)  0.856 (.113)  0.635 (.399)  0.142 (.718)  0.524 (.450)  0.558 (.383) |  | **0.910 (.001)**  0.547 (.208)  **1.160 (.000)**  **0.933 (.030)**  **1.114 (.000)**  **0.801 (.044)**  0.354 (.333) | **0.680 (.005)**  0.131 (.736)  **0.764 (.005)**  0.619 (.107)  **0.588 (.003)**  0.522 (.139)  0.069 (.831) |
| Health consciousness |  |  | 0.051 (.856) |  |  | 0.177 (.215) |
| Health information orientation |  |  | **1.155 (.000)** |  |  | **1.467 (.000)** |
| eHealth literacy |  |  | **-0.563 (.030)** |  |  | -0.204 (.122) |
| *Effect size (R^2^*) | *0.004* | *0.017* | *0.048* | *0.031* | *0.143* | *0.336* |

^1^ N= 800

^2^ *P* < .05

^3^ *P* < .01
