# Supplementary material for: Influence of the Business Revenue, Recommendation, and Provider Models on Mobile Health App Adoption: Three-Country Experimental Vignette Study
Source: JMIR Mhealth Uhealth. 2020 Jun 4;8(6):e17272. doi: 10.2196/17272 (PMC7303831; doi:10.2196/17272)
Supplement: Multimedia Appendix 6 [file mhealth_v8i6e17272_app6.docx]

Multimedia Appendix 6

Linear regression analyses with willingness to pay and intention to download for the data collection models in the Netherlands

|  | The Netherlands | | | | | |
| --- | --- | --- | --- | --- | --- | --- |
|  | WTP | | | Intention to Download | | |
|  | Model 1 | Model 2^2^ | Model 3^2^ | Model 1^3^ | Model 2^3^ | Model 3^3^ |
| Constant | **5.803 (.000)** | **4.644 (.017)** | **4.935 (.021)** | **6.175 (.000)** | **4.504 (.000)** | **3.364 (.011)** |
| Data collection models (no protection is ref) | **1.855 (.000)** | **1.934 (.000)** | **1.975 (.000)** | **1.382 (.000)** | **1.465 (.000)** | **1.600 (.000)** |
| Gender (male is ref) |  | -0.493 (.289) | -0.470 (.312) |  | 0.079 (.780) | 0.062 (.687) |
| Age |  | 0.012 (.434) | 0.011 (.494) |  | 0.006 (.541) | 0.007 (.493) |
| Education (student is ref)  High school  Some university  University  Postgraduate  Employed (yes is ref)  Financial Status (mostly is ref)  From time to time  Almost never |  | 1.535 (.351)  1.689 (.301)  1.669 (.297)  1.298 (.427)  -0.096 (.852)  0.226 (.793)  0.133 (.857) | 1.634 (.324)  1.769 (.264)  1.675 (.297)  1.308 (.425)  -0.045 (.932)  0.149 (.864)  0.113(.879) |  | 1.401 (.179)  1.096 (.289)  1.419 (.162)  1.185 (.252)  -0.357 (.253)  0.163 (.750)  0.459 (.300) | 0.948 (.358)  0.654 (.523)  1.069 (.286)  0.904 (.375)  -0.451 (.145)  0.276 (.585)  0.652 (.138) |
| Health consciousness |  |  | 0.235 (.444) |  |  | 0.081 (.657) |
| Health information orientation |  |  | 0.007 (.980) |  |  | **0.591 (.003)** |
| eHealth literacy |  |  | -0.349 (.153) |  |  | -0.041 (.778) |
| *Effect size (R^2^*) | *0.043* | *0.057* | *0.064* | *0.060* | *0.075* | *0.112* |

^1^ N= 380

^2^ *P* < .05

^3^ *P* < .01
