# Supplementary material for: Influence of the Business Revenue, Recommendation, and Provider Models on Mobile Health App Adoption: Three-Country Experimental Vignette Study
Source: JMIR Mhealth Uhealth. 2020 Jun 4;8(6):e17272. doi: 10.2196/17272 (PMC7303831; doi:10.2196/17272)
Supplement: Multimedia Appendix 7 [file mhealth_v8i6e17272_app7.docx]

Multimedia Appendix 7

Linear regression analyses with willingness to pay and intention to download for the recommendation models in Spain

|  | Spain | | | | | |
| --- | --- | --- | --- | --- | --- | --- |
|  | WTP | | | Intention to Download | | |
|  | Model 1 | Model 2 | Model 3^2^ | Model 1^2^ | Model 2^3^ | Model 3^3^ |
| Constant | **3.882 (.000)** | **4.918 (.002)** | 2.178 (.228) | **6.627 (.000)** | **7.850 (.000)** | **3.305 (.000)** |
| Recommendation (patient association is ref) | 0.516 (.174) | 0.460 (.227) | 0.497 (.191) | **0.382 (.043)** | 0.278 (.131) | **0.325 (.057)** |
| Gender (male is ref) |  | **-0.867 (.024)** | **-0.926 (.016)** |  | **-0.444 (.017)** | **-0.544 (.002)** |
| Age |  | -0.013 (.378) | -0.008 (.584) |  | **-0.037 (.000)** | **-0.032 (.000)** |
| Education (student is ref)  High school  Some university  University  Postgraduate  Employed (yes is ref)  Financial Status (mostly is ref)  From time to time  Almost never |  | -0.040 (.971)  -0.334 (.774)  0.118 (.916)  0.697 (.561)  0.638 (.163)  0.117 (.839)  0.305 (.582) | -0.128 (.909)  -0.180 (.876)  0.185 (.867)  0.647 (.588)  0.503 (.271)  0.101 (.861)  0.294 (.598) |  | 0.268 (.623)  -0.276 (.623)  0.230 (.668)  0.588 (.310)  **0.573 (.010)**  0.486 (.081)  0,036 (.893) | 0.0749 (.883)  -0.131 (.801)  0.229 (.646)  0.434 (.420)  0.360 (.081)  0.476 (.067)  0.001 (.996) |
| Health consciousness |  |  | -0.070 (.826) |  |  | 0.204 (.153) |
| Health information orientation |  |  | **0.905 (.010)** |  |  | **0.972 (.000)** |
| eHealth literacy |  |  | -0.067 (.810) |  |  | 0.124 (.319) |
| *Effect size (R^2^*) | *0.002* | *0.019* | *0.034* | *0.005* | *0.066* | *.212* |

^1^ N= 800

^2^ *P* < .05

^3^ *P* < .01
