# Supplementary material for: Influence of the Business Revenue, Recommendation, and Provider Models on Mobile Health App Adoption: Three-Country Experimental Vignette Study
Source: JMIR Mhealth Uhealth. 2020 Jun 4;8(6):e17272. doi: 10.2196/17272 (PMC7303831; doi:10.2196/17272)
Supplement: Multimedia Appendix 8 [file mhealth_v8i6e17272_app8.docx]

Multimedia Appendix 8

Linear regression analyses with willingness to pay and intention to download for the recommendation models in Germany

|  | Germany | | | | | |
| --- | --- | --- | --- | --- | --- | --- |
|  | WTP | | | Intention to Download | | |
|  | Model 1^2^ | Model 2^3^ | Model 3^2^ | Model 1^2^ | Model 2^3^ | Model 3^3^ |
| Constant | **3.713 (.000)** | **3.539 (.000)** | 1.764 (.112) | **5.750 (.000)** | **5.834 (.000)** | **1.845 (.009)** |
| Recommendation (patient association is ref) | **0.738 (.015)** | **0.615 (.042)** | 0.503 (.091) | **0.550 (.013)** | 0.375 (.076) | 0.217 (.247) |
| Gender (male is ref) |  | -0.033 (.914) | -0.165 (.578) |  | **-0.482 (.022)** | **-0.720 (.000)** |
| Age |  | -0.021 (.052) | **-0.023 (.031)** |  | **-0.027 (.000)** | **-0.030 (.000)** |
| Education (student is ref)  High school  Some university  University  Postgraduate  Employed (yes is ref)  Financial Status (mostly is ref)  From time to time  Almost never |  | 0.297 (.444)  1.076 (.087)  **1.200 (.007)**  0.830 (.181)  0.529 (.099)  0.124 (.829)  0.227 (.669) | 0.173 (.652)  0.852 (.169)  **1.058 (.016)**  0.708 (.249)  0.276 (.390)  -0.005 (.993)  0.108 (.836) |  | **0.934 (.001)**  0.831 (.060)  **1.199 (.000)**  **1.144 (.009)**  **1.071 (.000)**  0.567 (.159)  0.187 (.614) | **0.684 (.005)**  0.396 (.311)  **0.776 (.005)**  **0.775 (.045)**  **0.525 (.009)**  0.286 (.423)  -0.105 (.750) |
| Health consciousness |  |  | 0.208 (.360) |  |  | 0.188 (.189) |
| Health information orientation |  |  | **1.048 (.000)** |  |  | **1.481 (.000)** |
| eHealth literacy |  |  | **-0.570 (.007)** |  |  | -0.155 (.244) |
| *Effect size (R^2^*) | *0.007* | *0.036* | *0.076* | *0.008* | *0.115* | *0.312* |

^1^ N= 800

^2^ *P* < .05

^3^ *P* < .01
