# Supplementary material for: Influence of the Business Revenue, Recommendation, and Provider Models on Mobile Health App Adoption: Three-Country Experimental Vignette Study
Source: JMIR Mhealth Uhealth. 2020 Jun 4;8(6):e17272. doi: 10.2196/17272 (PMC7303831; doi:10.2196/17272)
Supplement: Multimedia Appendix 9 [file mhealth_v8i6e17272_app9.docx]

Multimedia Appendix 9

Linear regression analyses with willingness to pay and intention to download for the recommendation models in the Netherlands

|  | The Netherlands | | | | | |
| --- | --- | --- | --- | --- | --- | --- |
|  | WTP | | | Intention to Download | | |
|  | Model 1^2^ | Model 2 | Model 3 | Model 1^2^ | Model 2 | Model 3^3^ |
| Constant | **5.983 (.000)** | 4.874 (.065) | 4.572 (.114) | **5.504 (.000)** | **3.531 (.003)** | **2.494 (.049)** |
| Recommendation (patient association is ref) | **1.502 (.014)** | **1.680 (.007)** | **1.833 (.003)** | **0.661 (.012)** | **0.726 (.007)** | **0.891 (.001)** |
| Gender (male is ref) |  | -0.697 (.627) | -0.675 (.282) |  | 0.173 (.529) | 0.159 (.553) |
| Age |  | 0.006 (.778) | 0.005 (.830) |  | 0.001 (.897) | 0.001 (.879) |
| Education (student is ref)  High school  Some university  University  Postgraduate  Employed (yes is ref)  Financial Status (mostly is ref)  From time to time  Almost never |  | 1.372 (.539)  1.936 (.382)  2.082 (.338)  2.325 (.296)  0.289 (.680)  0.217 (.850)  0.238 (.807) | 1.187 (.597)  1.701 (.445)  1.818 (.404)  2.109 (.343)  0.299 (.670)  0.186 (.871)  0.349 (.721) |  | 1.624 (.108)  1.338 (.182)  1.468 (.136)  1.531 (.127)  -0.398 (.189)  0.449 (.366)  0.543 (.206) | 1.149 (.247)  0.854 (.385)  1.076 (.264)  1.221 (.213)  -0.487 (.102)  -0.487 (.102)  0.558 (.251) |
| Health consciousness |  |  | 0.295 (.477) |  |  | 0.108 (.540) |
| Health information orientation |  |  | 0.478 (.231) |  |  | **0.627 (.000)** |
| eHealth literacy |  |  | -0.536 (.108) |  |  | -0.180 (.202) |
| *Effect size (R^2^*) | *0.016* | *0.027* | *0.039* | *0.015* | *0.032* | *0.086* |

^1^ N= 380

^2^ *P* < .05

^3^ *P* < .01
