# Supplementary material for: Influence of the Business Revenue, Recommendation, and Provider Models on Mobile Health App Adoption: Three-Country Experimental Vignette Study
Source: JMIR Mhealth Uhealth. 2020 Jun 4;8(6):e17272. doi: 10.2196/17272 (PMC7303831; doi:10.2196/17272)
Supplement: Multimedia Appendix 10 [file mhealth_v8i6e17272_app10.docx]

Multimedia Appendix 10

Linear regression analyses with willingness to pay and intention to download for the provider models in Spain

|  | Spain | | | | | |
| --- | --- | --- | --- | --- | --- | --- |
|  | WTP | | | Intention to Download | | |
|  | Model 1 | Model 2 | Model 3^1^ | Model 1^3^ | Model 2^3^ | Model 3^3^ |
| Constant | **4.284 (.000)** | **4.601 (.006)** | 2.237 (.258) | **6.880 (.000)** | **9.036 (.000)** | **4.089 (.000)** |
| Provider (pharmaceutical company is ref) | 0.775 (.065) | 0.756 (.072) | 0.709 (.090) | **0.710 (.000)** | **0.753 (.000)** | **0.697 (.000)** |
| Gender (male is ref) |  | **-0.921 (.031)** | **-0.977(.022)** |  | -0.376 (.051) | **-0.488 (.006)** |
| Age |  | -0.004 (.804) | 0.004 (.829) |  | **-0.038 (.000)** | **-0.032 (.000)** |
| Education (student is ref)  High school  Some university  University  Postgraduate  Employed (yes is ref)  Financial Status (mostly is ref)  From time to time  Almost never |  | 0.093 (.940)  -0.012 (.993)  0.577 (.638)  0.996 (.452)  0.697 (.166)  0.491 (.441)  0.269 (.660) | 0.055 (.965)  0.188 (.883)  0.706 (.564)  0.980 (.458)  0.564 (.263)  0.493 (.439)  0.304 (.621) |  | -0.310 (.582)  -0.955 (.100)  -0.313 (.573)  0.031 (.959)  **0.594 (.009)**  0.252 (.381)  -0.218 (.430) | -0.508 (.327)  -0.793 (.138)  -0.306 (.549)  -0.144 (.795)  0.357 (.090)  0.261 (.327)  -0.228 (.376) |
| Health consciousness |  |  | -0.387 (.271) |  |  | 0.097 (.507) |
| Health information orientation |  |  | **1.128 (.004)** |  |  | **1.100 (.000)** |
| eHealth literacy |  |  | -0.134 (.661) |  |  | 0.170 (.185) |
| *Effect size (R^2^*) | *0.004* | *0.024* | *0.035* | *0.016* | *0.086* | *0.231* |

^1^ N= 800

^2^ *P* < .05

^3^ *P* < .01
