# Supplementary material for: Influence of the Business Revenue, Recommendation, and Provider Models on Mobile Health App Adoption: Three-Country Experimental Vignette Study
Source: JMIR Mhealth Uhealth. 2020 Jun 4;8(6):e17272. doi: 10.2196/17272 (PMC7303831; doi:10.2196/17272)
Supplement: Multimedia Appendix 11 [file mhealth_v8i6e17272_app11.docx]

Multimedia Appendix 11

Linear regression analyses with willingness to pay and intention to download for the provider models in Germany

|  | Germany | | | | | |
| --- | --- | --- | --- | --- | --- | --- |
|  | WTP | | | Intention to Download | | |
|  | Model 1 | Model 2^2^ | Model 3^3^ | Model 1^3^ | Model 2^3^ | Model 3^3^ |
| Constant | **2.520 (.000)** | **3.385 (.001)** | **2.375 (.047)** | **5.743 (.000)** | **6.513 (.000)** | **3.031 (.000)** |
| Provider (pharmaceutical company is ref) | 0.010 (.975) | 0.071 (.820) | 0.085 (.782) | **0.783 (.000)** | **0.871 (.000)** | **0.836 (.000)** |
| Gender (male is ref) |  | **-**0.081 (.794) | -0.180 (.556) |  | -0.368 (.208) | -0.587 (.002) |
| Age |  | -0.031 (.005) | **-0.031 (.004)** |  | **-0.028 (.000)** | **-0.030 (.000)** |
| Education (student is ref)  High school  Some university  University  Postgraduate  Employed (yes is ref)  Financial Status (mostly is ref)  From time to time  Almost never |  | 0.488 (.223)  0.762 (.240)  0.754 (.099)  0.784 (.220)  0.293 (.374)  0.114 (.848)  0.232 (.670) | 0.411 (.299)  0.573 (.369)  0.667 (.141)  0.757 (.230)  0.053 (.871)  0.010 (.987)  0.141 (.793) |  | **0.751 (.005)**  0.608 (.163)  **1.131 (.000)**  **0.958 (.026)**  **0.944 (.000)**  0.316 (.427)  -0.077 (.834) | **0.532 (.027)**  0.200 (.604)  **0.758 (.006)**  0.660 (.085)  **0.418 (.037)**  0.046 (.891)  -0.354 (.275) |
| Health consciousness |  |  | -0.059 (.800) |  |  | 0.130 (.359) |
| Health information orientation |  |  | **1.215 (.000)** |  |  | **1.563 (.000)** |
| eHealth literacy |  |  | **-0.663 (.002)** |  |  | **-0.299 (.023)** |
| *Effect size (R^2^*) | *0.004* | *0.022* | *0.063* | *0.016* | *0.109* | *0.299* |

^1^ N= 800

^2^ *P* < .05

^3^ *P* < .01
