# Supplementary material for: Influence of the Business Revenue, Recommendation, and Provider Models on Mobile Health App Adoption: Three-Country Experimental Vignette Study
Source: JMIR Mhealth Uhealth. 2020 Jun 4;8(6):e17272. doi: 10.2196/17272 (PMC7303831; doi:10.2196/17272)
Supplement: Multimedia Appendix 12 [file mhealth_v8i6e17272_app12.docx]

Multimedia Appendix 12

Linear regression analyses with willingness to pay and intention to download for the provider models in the Netherlands

|  | The Netherlands | | | | | |
| --- | --- | --- | --- | --- | --- | --- |
|  | WTP | | | Intention to Download | | |
|  | Model 1^3^ | Model 2 | Model 3^2^ | Model 1^3^ | Model 2^3^ | Model 3^3^ |
| Constant | **4.965 (.000)** | 3.226 (.155**)** | 3.028 (.222) | **5.380 (.000)** | **3.751 (.002)** | **2.831 (.031)** |
| Provider (pharmaceutical company is ref) | **1.317 (.009)** | **1.389 (.006)** | **1.497 (.004)** | **0.949 (.000)** | **0.971 (.000)** | **1.094 (.000)** |
| Gender (male is ref) |  | -0.696 (.179) | -0.662 (.201) |  | 0.145 (.607) | 0.133 (.631) |
| Age |  | 0.013 (.473) | 0.011 (.542) |  | 0.006 (.542) | 0.006 (.520) |
| Education (student is ref)  High school  Some university  University  Postgraduate  Employed (yes is ref)  Financial Status (mostly is ref)  From time to time  Almost never |  | 1.783 (.362)  2.496 (.200)  1.902 (.321)  2.518 (.197)  0.507 (.376)  0.394 (.674)  0.248 (.758) | 1.772 (.366)  2.454 (.210)  1.761 (.358)  2.433 (.212)  0.563 (.328)  0.326 (.728)  0.260 (.748) |  | 1.198 (.246)  1.208 (.239)  1.088 (.280)  1.073 (.295)  -0.261 (.400)  0.041 (.936)  0.332 (.450) | 0.823 (.423)  0.834 (.413)  0.783 (.432)  0.831 (.413)  -0.332 (.280)  0.128 (.800)  0.496 (.256) |
| Health consciousness |  |  | 0.414 (.218) |  |  | 0.102 (.575) |
| Health information orientation |  |  | 0.164 (.610) |  |  | **0.459 (.008)** |
| eHealth literacy |  |  | -0.471 (.084) |  |  | -0.099 (.497) |
| *Effect size (R^2^*) | *0.018* | *0.024* | *0.050* | *0.030* | *0.037* | *0.070* |

^1^ N= 380

^2^ *P* < .05

^3^ *P* < .01
